# From “quantifying the child” to “supporting the caregiver”: a paradigm evaluation and ethical pathway selection for AI applications in child development

**DOI:** 10.3389/fpsyg.2026.1682555

**Published:** 2026-02-17

**Authors:** Yan Xu, Hang Chen, Mu-Mu Wei, Yi Zhang, Hong-Tao Cui, Fang Liu

**Affiliations:** 1 Department of Pediatrics, First Affiliated Hospital, Henan University of Chinese Medicine, Zhengzhou, Henan, China; 2 School of Pediatrics, Henan University of Chinese Medicine, Zhengzhou, Henan, China; 3 Department of Pediatrics, Chongqing Traditional Chinese Medicine Hospital, The First Affiliated Hospital of Chongqing University of Chinese Medicine, Chongqing, China; 4 Department of Endocrinology and Metabolism, Chongqing Traditional Chinese Medicine Hospital, The First Affiliated Hospital of Chongqing University of Chinese Medicine, Chongqing, China

**Keywords:** artificial intelligence, caregiver support, child development, paradigm evaluation, quantified self, technology ethics

## Abstract

**Background:**

Artificial Intelligence (AI) is rapidly reshaping pediatric care. While AI-driven clinical screening tools have demonstrated significant value in the early identification of neurodevelopmental risks (e.g., dyslexia, autism), a parallel trend of continuous, consumer-grade quantification of neurotypical children is emerging.

**Problem:**

This paper critically evaluates the “Quantified Child” paradigm—defined as the use of consumer technologies for continuous physiological and behavioral tracking. We argue that unlike targeted clinical interventions, this pervasive surveillance approach carries systemic risks: it may induce “technoference” in parent-child interactions, amplify caregiver performance anxiety, and trigger iatrogenic risks through false-positive labeling.

**Proposal:**

Drawing on developmental science, we propose shifting to a “Supporting the Caregiver” paradigm for non-clinical settings. In this model, AI functions as an administrative assistant to automate family logistics and reduce cognitive load, rather than a digital intermediary for monitoring the child.

**Mechanism:**

We posit that reducing caregiver stress and saving effective time serve as crucial proximal mediators. By improving the caregiver's psychological well being, AI indirectly protects the quality of responsive parenting, which is the definitive driver of positive child developmental outcomes in early childhood.

**Conclusion:**

A paradigm shift from direct child quantification to caregiver support offers a more robust and ethical technological pathway, ensuring that AI serves to enrich, rather than displace, the human connections essential for early development.

## 1 Introduction

Artificial Intelligence (AI) technology is rapidly penetrating the ecosystem of child development. From intelligent baby monitors to developmental tracking apps, digital tools are continuously reshaping parenting practices. Crucially, however, we must distinguish between two fundamentally different applications of AI in this domain.

On one hand, AI-driven clinical screening tools have demonstrated immense value in the early identification of neurodevelopmental risks (e.g., dyslexia, autism) within professional settings (Achenie et al., 2019; Svaricek et al., 2025). These tools are beneficial because they are targeted, episodic, and evidence-based. However, a concerning parallel trend is the generalization of this medical logic into the daily lives of neurotypical children through consumer-grade technologies. We term this emerging paradigm the “Quantified Child,” which aims to achieve fine-grained tracking and optimization of developmental trajectories by continuously collecting data on children's physiological and behavioral performance.

A profound tension exists between this technological path of “continuous quantification” and the core principles of developmental science, particularly in early childhood (0–6 years). Long-term evidence from developmental science repeatedly emphasizes that high-quality, stable parent-child interactions and a supportive family environment are the cornerstones of children's social-emotional competence and overall well being (Balaj et al., 2021; Egeland et al., 1990). This evidence suggests that the primary driver of development is the quality of the relationship, not the quantity of data. Consequently, any technology that inserts itself as a “digital intermediary” between parent and child—potentially causing “technoference” or performance anxiety—must be critically examined.

Existing research has fully confirmed that caregivers' psychological states and family adversity are significant risk factors, but it has also demonstrated that the quality of family interaction is significantly malleable and intervenable (Adjei et al., 2024). This fact provides a pivotal perspective for rethinking technology: the value of AI may not lie in directly “optimizing” the child, but in indirectly “supporting” the caregiver.

Therefore, the objectives of this paper are twofold. First, based on an integrated evidence base, we conduct a structured risk assessment of the “Quantified Child” paradigm in the consumer sector, arguing that its inherent risks—such as the disruption of responsive parenting—outweigh its benefits for neurotypical children. Second, we propose an alternative paradigm centered on “Supporting the Caregiver.” We argue that shifting the technological focus from monitoring children to reducing caregiver stress and logistical burden represents a more ethical and effective path, aligning technological capabilities with the biological realities of early child development.

## 2 Systemic risks of the “quantified child” paradigm

AI applications that directly source data from children for continuous quantitative assessment are inherently designed to produce a series of foreseeable, systemic negative effects. These effects are not “side effects” that can be eliminated through technological-optimization, but are innate flaws of the paradigm, mainly reflected in the following four aspects.

### 2.1 Increased caregiver psychological stress

By continuously providing a comparison of a child's individual data with standardized norms, the feedback mechanism of quantitative AI inevitably intensifies the performance-based pressure on caregivers in a sociocultural context of high-investment parenting. Research has clearly indicated that parenting stress is a key variable affecting the quality of family relationships and children's behavioral and emotional outcomes, and that psychological support for parents can effectively reduce their stress levels, proving the intervenable nature of this pathway (Egeland et al., 1990; Mo et al., 2024). When a technological system constantly shapes parents' attention and parenting goals with alerts of “deviation from the norm,” parents are highly likely to invest their limited time and energy in the short-term “optimization” of data metrics. This data-driven anxiety, through emotional exhaustion and controlling interactions, ultimately reduces the quality of parent-child interaction, thus posing an indirect but continuous adverse effect on the child's emotional security.

### 2.2 Narrowing of key developmental areas

The effectiveness of algorithms is naturally limited to variables that are easy to operationalize and digitize, such as a child's vocabulary size, sleep duration, or screen time. However, classic theories of developmental science, such as the ecological systems theory, repeatedly emphasize the decisive role of multi-level systems and relational processes in development. These factors include the quality of parent-child interaction, family cohesion, and community support, which are inherently difficult to quantify precisely (Brooks-Gunn et al., 2000). When the resources and attention of parents and society are excessively absorbed by a few measurable indicators, it may come at the cost of time spent on high-quality interactions, joint reading, and outdoor activities. This phenomenon can be termed “measurability bias,” and it leads to an increase in parenting opportunity costs and a severe narrowing of the developmental focus.

### 2.3 Introduction of labeling and iatrogenic risks

In complex real-world scenarios, algorithmic false positives and uncertainty are technologically inherent and difficult to eradicate. A wrong label, even if it is just a probabilistic risk alert, can have a profound and negative “imprinting effect” on a child's self-concept and a parent's nurturing decisions. Evidence from the field of pediatric screening clearly indicates that false-positive results can significantly increase parental anxiety and trigger a chain of unnecessary medical consultations and over-interventions, which constitutes a typical iatrogenic risk (O'Leary et al., 2024). When a probabilistic risk alert is misinterpreted by a non-professional end-user (the parent) as a definitive diagnostic label, it can fundamentally change their interaction patterns with the child and the allocation of family resources, ultimately amplifying avoidable medical burdens and psychological stress. For example, the American Academy of Pediatrics (AAP) explicitly stated in its 2022 policy statement that it does not recommend consumer-grade cardiorespiratory monitors as a means to reduce the risk of Sudden Infant Death Syndrome (SIDS), with the core logic being to prevent the misleading sense of security they provide from replacing truly effective evidence-based guidance and environmental modifications (Moon et al., 2022).

### 2.4 Disruption of responsive parenting: the “technoference” effect

Perhaps the most profound risk is the interference with responsive parenting—the cornerstone of early childhood development. This mechanism relies on the caregiver's sensitive, timely, and contingent feedback to the child's signals (Olson et al., 1990). However, the “Quantified Child” paradigm inserts a digital interface between parent and child. Recent empirical studies on “technoference” (technology-based interference) suggest that frequent checking of devices disrupts the natural “serve and return” interaction patterns essential for brain development (McDaniel and Radesky, 2018a,b). When caregivers rely on a “digital intermediary” to interpret their child's needs (e.g., checking an app to see if the baby is hungry rather than reading the baby's cues), it risks “de-skilling” their intuitive parenting abilities. The caregiver's attention is fractured, shifting from the child's face to the data screen, thereby degrading the quality of the immediate, embodied connection that is critical for secure attachment.

## 3 The alternative paradigm: AI as a caregiver support system

Based on the systematic deconstruction of the risks associated with continuous quantification—particularly the risks of technoference and iatrogenic anxiety—this paper argues that the application of consumer-grade AI in early childhood must undergo a fundamental shift. We propose an alternative paradigm: moving from “Quantifying the Child” to “Supporting the Caregiver.”

The theoretical foundation of this paradigm shift lies in the long-standing consensus of developmental science: high-quality parent-child interaction and a supportive family environment are the strongest predictors of a child's healthy development. As emphasized by ecological systems theory, the synergistic effects of multi-level systems—including parents, schools, and communities—jointly shape an individual's developmental trajectory, rather than isolated biological metrics (Brooks-Gunn et al., 2000).

Crucially, this paradigm does not ignore the child; rather, it prioritizes the indirect pathway to child well being. Solid intervention evidence supports this logic: Meta-analyses show that interventions focused on parents (such as parent training) can significantly reduce parenting stress (Standardized Mean Difference, SMD = −0.38, 95% CI: −0.49 to −0.27) and improve the quality of parent-child interaction (Shah et al., 2022). Furthermore, technology-assisted interventions, such as video feedback, have been proven to effectively enhance caregiver sensitivity (SMD = 0.34, 95% CI: 0.20–0.49), directly translating into better attachment security for the child (O'Hara et al., 2019).

Therefore, the primary value of AI should not be to monitor the child as a passive subject, but to empower the adult as an active agent. By automating administrative burdens and providing just-in-time, stress-reducing support, AI can protect the “relational space” needed for responsive parenting.

From an ethical perspective, shifting the point of technological intervention from the vulnerable child to the autonomous adult (the caregiver) adheres more strictly to the “do not harm” principle. This approach avoids the risks of labeling and medicalizing normal childhood variations while maximizing the benefits of evidence-based environmental optimization. Thus, the “Support the Caregiver” paradigm is not merely a risk-mitigation strategy, but a scientifically grounded pathway to optimize child outcomes by nurturing the nurturer.

## 4 Operational definition and boundaries of the alternative paradigm

The core principle of this alternative paradigm is that AI should be designed to support the human caregiving system rather than to replace human judgment with algorithmic data. To ensure ethical application, it is essential to establish strict operational boundaries based on the setting of use, distinguishing clearly between consumer-oriented home environments and professional clinical settings.

### 4.1 Application for parents: an administrative and logistical support system

For parents of neurotypical children, AI should function as a “Family Affairs Assistant” aimed at reducing cognitive load and time costs, rather than a “Developmental Monitor.” The primary objective is to liberate parents from tedious transactional work—such as family meal planning, schedule coordination, and the personalized retrieval of reliable parenting knowledge—allowing them to reinvest their time in high-quality, face-to-face interactions. By automating these logistical tasks, technology serves to minimize “technoference” and protect the relational space between parent and child.

Conversely, this paradigm strictly prohibits the collection, analysis, or scoring of a child's physiological or behavioral data in a consumer context. The system should not include functions for continuous child development tracking or peer ranking, which often serve to amplify parental anxiety. Furthermore, in alignment with the evidence-based recommendations of the American Academy of Pediatrics (AAP), this paradigm explicitly opposes the promotion of consumer-grade wearable devices for routine vital sign monitoring in healthy infants, as these tools often provide a misleading sense of security and trigger unnecessary iatrogenic alarm (Moon et al., 2022).

### 4.2 Application for clinicians: a deeply integrated decision support system

In contrast to the consumer domain, the “Support the Caregiver” paradigm within professional settings extends to supporting the professional caregiver—the pediatrician or therapist. In this specific context, quantification and automated screening are both permissible and highly valuable, provided they are designed to enhance rather than replace clinical expertise.

First, AI can serve as a powerful tool for pre-visit screening and information integration. By analyzing structured data—such as standardized developmental questionnaires or targeted screening tools for conditions like dyslexia (e.g., EarlyBird)—AI can efficiently identify high-risk markers before a consultation. This application leverages the computational strengths of AI to ensure early identification, directly addressing the critical need for timely intervention in potential neurodevelopmental disorders.

Second, AI should be deeply integrated into the clinical workflow to function as an efficiency partner. By automating administrative tasks—such as extracting key information from Electronic Health Records (EHR), transcribing consultations in real-time, and drafting standardized medical documentation—AI allows clinicians to redirect their attention from the computer screen back to the patient. However, it is imperative that the physician remains the “human-in-the-loop.” All AI-generated insights must be presented strictly as decision support, ensuring that the final interpretation of a child's developmental status relies on professional human judgment rather than algorithmic output alone.

## 5 Validation standards for the alternative paradigm's effectiveness

The evaluation of AI tools under the “Support the Caregiver” paradigm requires a fundamental shift in validation methodology. We acknowledge that the ultimate “gold standard” for any pediatric intervention remains the improvement of child developmental outcomes. However, obtaining these outcomes through continuous digital surveillance introduces the very risks of labeling and technoference we seek to avoid. Therefore, we propose a hierarchical validation framework that distinguishes between proximal mediators (caregiver metrics) and distal outcomes (child metrics), verifying the efficacy of AI through an indirect but causal pathway.

The primary level of validation should focus on proximal mediators: specifically, “effective time saved” and “reduction in caregiver physiological and psychological stress.” Unlike the “Quantified Child” paradigm which views these metrics as secondary user experience factors, this new paradigm posits them as critical clinical mechanisms. The logic is that by offloading cognitive drudgery and logistical burdens, AI preserves the caregiver's emotional capacity. Effectiveness should be measured by the reallocation of this saved time: validation must confirm that the time “freed” by AI is not displaced by screen time, but is reinvested in high-quality, face-to-face parent-child interactions.

The secondary, and more critical, level of validation is the demonstrable improvement in distal child development outcomes. We argue that while the AI tool itself should not monitor the child, the scientific validation of the tool must verify its downstream benefits. Researchers should employ Randomized Controlled Trials (RCTs) using a mediation analysis model to prove that the reduction in parental stress (facilitated by AI) statistically mediates improvements in the child's secure attachment and social-emotional competence. For instance, standardized clinical assessments (administered by professionals, not apps) should demonstrate that children in families using “Supportive AI” exhibit better developmental trajectories compared to controls, not because the machine optimized the child, but because the machine successfully nurtured the family environment.

## 6 Discussion

The central argument of this paper is that the application of AI in early childhood development faces a fundamental paradigm choice. This is not merely a technical optimization problem, but a question of values: should technology serve to “Quantify the Child” or to “Support the Caregiver”?

### 6.1 The fallacy of generalized quantification

The “Quantified Child” paradigm represents an extension of the engineering mindset—pursuing efficiency and certainty—into the organic realm of parenting. While this reductionist approach promises objective control, it fundamentally conflicts with the holistic and relational nature of early childhood. Crucially, our critique focuses on the generalization of this paradigm into the consumer market for neurotypical children. A common counterargument, often raised by proponents of precision medicine, is that early quantitative screening is essential for identifying risks in neurodevelopmental disorders. We fully agree with this premise. However, the value of professional, targeted screening tools (e.g., for dyslexia or autism) does not justify the continuous, invasive surveillance of healthy children in their homes. The danger lies in the “medicalization of everyday life,” where the logic of the clinic—constant monitoring for defects—displaces the logic of the home, which should be centered on unconditional acceptance and connection.

### 6.2 The mediation mechanism: supporting the parent to save the child

In contrast, the “Supporting the Caregiver” paradigm represents a philosophy of prudent technological application. It acknowledges that for the first 6 years of life, the most sophisticated “developmental support system” is not an algorithm, but a responsive human adult. Therefore, the mechanism of this paradigm is indirect but potent: by acting as a “buffer” against administrative stress and logistical chaos, AI protects the caregiver's psychological resources. *This aligns with the “Mediation Model” of family intervention: AI reduces parental stress, which in turn reduces negative “technoference” behaviors, ultimately preserving the quality of parent-child interaction that drives child outcomes*.

### 6.3 Implementation challenges and governance

Implementing this paradigm faces significant challenges, primarily from business models incentivized by user engagement and data collection. To correct this market failure, we need a multi-level governance framework ensuring the Safety, Equity, Effectiveness, and Trustworthiness (SEET principles) of technology deployment (Rozenblit et al., 2025). Key governance priorities must include: *(1) Strict Data Minimization*: Prohibiting the collection of unnecessary child data in non-clinical apps; *(2) Human-in-the-Loop*: Ensuring that all AI decisions involving child welfare maintain human professionals as the final authority; and *(3) Post-Market Surveillance*: Establishing mechanisms to address model drift and bias over time (Husain et al., 2024). Furthermore, voluntary certification frameworks could incentivize the development of “privacy-enhancing” applications, similar to standards emerging in wearable body-fluid analysis (Brasier et al., 2024), but adapted for the digital well being of the family.

### 6.4 Future research directions

Future research should focus on empirically testing this alternative paradigm. We call for Randomized Controlled Trials (RCTs) that compare “Quantified Child” apps against “Caregiver Support” tools. *Crucially, these studies must measure the “Technoference Effect”: tracking not just whether the app works, but whether using the app physically and attentionally displaces parent-child interaction*. Only by including these ecological metrics can we truly assess the net impact of AI on family well being.

## 7 Conclusion

This paper has conducted a systematic risk assessment of the mainstream application of artificial intelligence in early childhood development. Our analysis reveals that the “Quantified Child” paradigm—when applied to consumer-grade surveillance of neurotypical children—harbors systemic flaws. It risks increasing caregiver performance anxiety, narrowing developmental focus, and inducing “technoference” that disrupts core parent-child interaction mechanisms.

Accordingly, we conclude that a responsible path for AI requires a fundamental paradigm shift. The industry must pivot from developing tools that “watch” the child to developing tools that “serve” the caregiver. By strictly defining AI as an auxiliary administrative support system, we can harness its computational power to reduce the burdens of modern parenting without sacrificing the human connection. Ultimately, the goal of AI in early childhood should not be to optimize data points, but to protect the unquantifiable, interpersonal moments that constitute the foundation of human development.
